# Influence of a Major Mountainous Landscape Barrier (Mount Cameroon) on the Spread of Metabolic (*GSTe2*) and Target-Site (*Rdl*) Resistance Alleles in the African Malaria Vector *Anopheles funestus* †

**DOI:** 10.3390/genes11121492

**Published:** 2020-12-11

**Authors:** Nathalie Amvongo-Adjia, Jacob M. Riveron, Flobert Njiokou, Samuel Wanji, Charles S. Wondji

**Affiliations:** 1 Department of Animal Biology and Physiology, Faculty of Science, University of Yaoundé 1, P.O. Box 812 Yaoundé, Cameroon; njiokouf@yahoo.com; 2 Research Foundation for Tropical Diseases and Environment (REFOTDE), University of Buea, P.O. Box 474 Buea, Cameroon; swanji@yahoo.fr; 3 Centre for Research in Infectious Diseases (CRID), LSTM Research Unit, P.O. Box 13591 Yaoundé, Cameroon; jacob.riveron_miranda@syngenta.com; 4 Medical Research Centre, Institute of Medical Research and Medicinal Plants Studies (IMPM), P.O. Box 13033 Yaoundé, Cameroon; 5 Department of Microbiology and Parasitology, University of Buea, P.O. Box 63 Buea, Cameroon; 6 Department of Vector Biology, Liverpool School of Tropical Medicine, Liverpool L35QA, UK

**Keywords:** malaria, *Anopheles funestus*, insecticide resistance, Mount Cameroon

## Abstract

Increased levels of insecticide resistance in major malaria vectors such as *Anopheles funestus* threaten the effectiveness of insecticide-based control programmes. Understanding the landscape features impacting the spread of resistance makers is necessary to design suitable resistance management strategies. Here, we examined the influence of the highest mountain in West Africa (Mount Cameroon; 4095 m elevation) on the spread of metabolic and target-site resistance alleles in *An*. *funestus* populations. Vector composition varied across the four localities surveyed along the altitudinal cline with major vectors exhibiting high parity rate (80.5%). *Plasmodium* infection rates ranged from 0.79% (*An. melas*) to 4.67% (*An. funestus*). High frequencies of *GSTe2^R^* (67–81%) and *Rdl^R^* (49–90%) resistance alleles were observed in *An. funestus* throughout the study area, with *GSTe2^R^* frequency increasing with altitude, whereas the opposite is observed for *Rdl^R^*. Patterns of genetic diversity and population structure analyses revealed high levels of polymorphisms with 12 and 16 haplotypes respectively for *GSTe2* and *Rdl*. However, the reduced diversity patterns of resistance allele carriers revealed signatures of positive selection on the two genes across the study area irrespective of the altitude. Despite slight variations associated with the altitude, the spread of resistance alleles suggest that control strategies could be implemented against malaria vectors across mountainous landscapes.

## 1. Introduction

Vector control of mosquitoes is a critical part of the global strategy to manage mosquito-associated diseases, and insecticides are the most important component in this effort. Four insecticide classes, namely organochlorines, organophosphates, carbamates and pyrethroids, are mainly recommended for use against adult mosquitoes [1]. Pyrethroids are the most widely used insecticides for indoor spraying against mosquitoes worldwide, owing to their efficacy and safety [2]. They are the main chemicals approved to treat mosquito nets [3,4], the main tool for human protection from malaria-carrying mosquitoes especially in Africa [1]. In the past, massive sprayings of insecticides greatly limited mosquito-borne diseases and even eradicated malaria in a few areas [5,6]. However, the widespread development of resistance in mosquitoes to the most commonly used insecticides negatively impacted the fight against mosquitoes in many areas [7]. This has resulted in a number of outbreaks of mosquito-borne diseases in recent years [8,9].

In Cameroon, malaria is the leading cause of morbidity and mortality, accounting for an estimated 6.2 million clinical cases and 12,500 deaths in 2018 [10]. *Anopheles funestus sensu stricto* (s.s.) is one of the four major malaria vectors in the country [11,12], being mostly prevalent in the Sudan savanna domain. However, this species had been found transmitting the *Plasmodium falciparum* malaria parasite in Cameroon highlands such as the Mount Cameroon region [13] and the locality of Santchou in western Cameroon [14]. The resistance profile of *An. funestus* s.s. has previously been explored for some populations, with multiple resistance to pyrethroids, dichlorodiphenyltrichloroethane (DDT) and carbamates reported in the localities of the sudano-sahelian [15,16,17] and forested [18,19] zones of Cameroon. Two point mutations, the *L119F* of the glutathione s-transferases epsilon 2 (*GSTe2*) gene conferring resistance to DDT and pyrethroids [20], and the *A296S* of the γ-aminobutyric acid (GABA) gene implicated in resistance to dieldrin (*Rdl*) [15], have been identified in *An. funestus* s.s. African populations. These markers have been associated with separate resistance mechanisms, respectively, the increased metabolic detoxification of insecticides, commonly known as metabolic resistance, and the decreased sensitivity of the target proteins on which an insecticide acts, so called target-site resistance [21]. Additionally, cytochromes *P450s* genes have been associated with pyrethroids resistance in the same vector [22,23].

Dispersal barriers such as mountains are known to have demographic, evolutionary and ecosystem-wide consequences [24]. Mountain ranges may delimit the vector-pathogen dynamics [25,26], as well as constitute potential barriers to the spread of invasive species [27]. The spread of resistance alleles is not uniform across the African continent, with *A296S-Rdl* and *L119F-GSTe2* being present in West and Central Africa but absent in southern Africa [15,20], whereas the CYP6P9a/b resistance alleles present an opposite distribution [28,29]. Presence of barriers to gene flow has been suggested to explain the restriction in the spread of these alleles [30,31]. Major landscape modifications such as Rift Valley have been suggested as the main continent-wide barrier to the spread of resistance alleles in *An*. *funestus* [30,31]. It remains unknown whether other major but local landscape modifications such as major mountain chains could also restrict the spread of these resistance alleles. The Mount Cameroon chain, with its highest peak at 4095 m above sea level (m a.s.l.), is the highest mountain in West Africa, and thus, constitutes a major landscape variation in the region with potential to impact patterns of gene flow between populations of species across this region. This potential influence on the spread of resistance alleles across the region remains unknown.

The Mount Cameroon region is also an area of prime importance to the Cameroon agriculture sector with industrial banana, rubber and palm plantations, activities that rely on the intensive use of agrochemicals likely to select resistance in local mosquito populations. This has already been shown in *An. coluzzii* and *An. gambiae*, which exhibit a high resistance level to pyrethroids in this region [32]. Thus, a similar resistance profile is expected in *An. funestus* vectors.

Understanding the influence of the high altitude of Mount Cameroon in the spread of resistance alleles could help to improve the efforts of malaria vector control and management of resistance. Here, we report the contribution to malaria transmission and the impact of Mount Cameroon on the spread of both GSTe2-based metabolic and Rdl-based target-site resistances by studying the genetic diversity of both genetic markers in *An. funestus* populations across Mount Cameroon altitudinal transect. Based on the analysis of *GSTe2* and *Rdl* markers, genotype and allele frequency distributions were compared between *An. funestus* s.s. populations collected in four localities across the Mount Cameroon region. Furthermore, we assessed signatures of selection for these genes and established a pattern of genetic structure between the four *An. funestus* s.s. populations. Beside the insecticide resistance profile, the *Plasmodium* infection rate of *An. funestus* s.s. from Mount Cameroon was also assessed.

## 2. Materials and Methods 

### 2.1. Study Site and Adult Mosquito Sampling

Mount Cameroon is the highest active volcano in western Africa, rising from the Atlantic Ocean at the Gulf of Guinea to an elevation of 4095 m a.s.l. The study was conducted in low, mid and high altitude areas within the lowland rain forest from sea level to 800 m elevation across the Mount Cameroon area (precisely in the Fako division, southwest region of Cameroon), having different landscape and environmental patterns [33]. Entomological surveys were restricted at this altitudinal range (from sea level to 800 m a.s.l.) due to previous surveys where the absence of *Anopheles* mosquitoes above 800 m elevation (precisely in the localities of Bokova and Bonakanda, situated between 800 and 870 m a.s.l.) was reported during four consecutive seasons [34].

**Figure 1:**
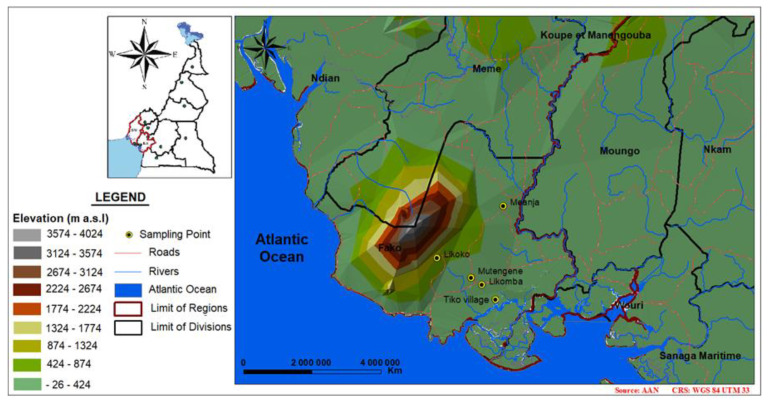
Map of the study area.

The localities surveyed during this study were: Tiko village (4°3′ N, 9°22′ E and elevation 9 m a.s.l.) and Likomba (4°5′ N, 9°20′ E and elevation 70 m a.s.l) located in the Tiko municipality and considered as lowlands (Tiko village and Likomba were further considered as a single collection site: Tiko), Mutengene (4°05′57″ N, 9°18′29″ E, altitude 220 m a.s.l.) and Meanja (4°16′ N, 9°23′ E, altitude 305 m a.s.l.), respectively located in the Tiko and Muyuka municipalities, were considered as mid altitude areas, and Likoko (4°8′41″ N, 9°13′38″ E, elevation 800 m a.s.l.), in the Buea municipality, was considered a highland area. Localities of Tiko, Likomba, Mutengene and Likoko follow an altitudinal transect on the southwest and west edge of the mountain, whereas Meanja was selected as an elevated area situated eastward to the mountain (outside the altitudinal transect) in order to assess if contrasting events occur across the mountain (Figure 1). The region has an equatorial climate modified by a double influence of the Atlantic Ocean and the mountain, and it is characterised by a unique rainy season from March to October, while the dry season goes from November to February [35]. Malaria transmission is perennial in low and mid altitude areas, whereas in the highlands it is seasonal with high transmission indices observed during the rainy season months [36,37]. *Anopheles gambiae*, *An. coluzzii* and *An. melas*, sibling species of the *An. gambiae* s.l. complex, and *An. funestus* s.s. are the prevalent malaria vectors [13,38,39].

Prior to the entomological field activities, authorisation was sought from the villages’ chiefs and sub-chiefs and quarters’ heads. The permission to collect mosquitoes from households was obtained from the household heads. Mosquito collectors were asked to sign a consent form before participating in night collections and were able to withdraw whenever they chose to do so. In addition, presumptive malaria prophylaxes were given to them at the end of collections.

Depending the surface area of selected localities, two to six neighbourhoods were visited for mosquito collections (Table S1). Female adult *Anopheles* mosquitoes were collected from the year 2010 to 2014 (2010: May; 2011: February-May-August-November; 2012: February-August-November; 2013: August-November; 2014: February) (Table S1), indoor and outdoor households between 06:00 PM to 06:00 AM using human landing catch technique as previously reported [39]. 

### 2.2. Species Identification and Plamodium Infection Rate

Female *Anopheles* that were caught were morphologically identified according to the key of [40], and ovaries were dissected for physiological age-grading [41]. For each specimen identified as belonging to either the *An. gambiae* complex or the *An. funestus* group, genomic DNA was extracted following the DNA extraction buffer (DEB) protocol [42] on whole mosquitoes. Sibling species of the *An. gambiae* complex were distinguished using conventional PCR [43] and PCR-RFLP [44] of the ribosomal intergenic spacer IGS of the nuclear rDNA gene, whereas members of the *An. funestus* group were characterised by amplifications of the internal transcribed spacer ITS2 [45]. Mosquito infection status was assessed using TaqMan assay as described by [46] and confirmed using multiplex single-round [47] and nested [48] PCR assays.

### 2.3. Genotyping of GSTe2 and Rdl Resistance Markers

*GSTe2* mutation associated with DDT and pyrethroids’ resistance in *An*. *funestus* was genotyped using an allele-specific PCR (AS-PCR) assay previously described by [49]. The same was done for the *Rdl* mutation conferring resistance to dieldrin as described by [50]. The primers used for these assays can be found as Table S2. Frequency distribution of *GSTe2* and *Rdl* genotypes and alleles were compared using the Chi-square (χ^2^) test, with statistical significance set at *p* < 0.05.

### 2.4. Polymorphism Analysis of Resistance Genes

#### 2.4.1. Genetic Variability of *An. funestus* s.s. across the Mount Cameroon Region Based on *GSTe2* and *Rdl* Full Gene Sequencing

Full-length sequences (all exons and introns) of *GSTe2* and *Rdl* were individually amplified for 40 samples (10 samples per locality each: Tiko, Mutengene, Meanja and Likoko) according to previous protocols [20,50]. Details of the primers used are provided in Table S2. Sample size determination was based on a previous assumption showing that for single nucleotide polymorphism makers, the most complete and unbiased representation of genetic diversity present in an individual can be obtained by incorporating a minimum of 10 individuals into the discovery data set, thus ensuring the discovery of both common and rare polymorphisms [51]. Successful amplicons were purified using the Exo-SAP clean up protocol (ThermoFisher Scientific, Santa Clara, CA, USA) and directly sequenced on both strands. Ninety other *An. funestus* DNA sequences, which have previously been analysed based on *GSTe2* mutation in a separate study [20], were also included for genetic variability analysis. These sequences were used in the present study to compare Mount Cameroon data with contemporary patterns of *GSTe2* gene flow specifically signs of directional selection between *An. funestus* populations across Africa [20]. These included *An. funestus* populations from Benin, Ghana, Malawi, Mozambique, Northern Cameroon and Uganda. The polymorphic positions were identified through a manual analysis of sequence chromatograms using BioEdit 7.2.5 [52] based on sequence differences in multiple alignments using ClustalW [53]. Genetic diversity parameters such as polymorphic sites S*, number of haplotypes h, haplotype diversity Hd, Synonymous mutations Syn, Non-synonymous mutations Nsyn, nucleotide diversity π, mean number of nucleotide differences k and Tajima D and Fu and Li F* neutrality estimates were computed using dnaSP 5.10 [54]. For each resistance gene, diversity parameters were useful to assess the level of biological diversity, the variations between and within *An. funestus* populations as well as the relationship between vector populations. Haplotype networks were then built using the TCS program [55,56] to assess the connection between haplotypes. The level of pairwise genetic differentiation were estimated using the K_ST_ statistics [57] as implemented in dnaSP 5.10. The significance of the K_ST_ estimates were assessed by permutation of subpopulation identities and re-calculating K_ST_ 10,000 times.

#### 2.4.2. Phylogenetic Trees of Haplotypes

Prior to the construction of the Maximum-Likelihood (ML) phylogenetic trees for *GSTe2* and *Rdl* haplotypes, the best-fit substitution model for each dataset was assessed based on the Bayesian Information Criterion (BIC) in MEGA 10.1.6 [58]. These analyses indicated that the Tamura 3-parameter, Kimura 2-parameter and Hasegawa-Kishino-Yano models best described *GSTe2* haplotype dataset of Mount Cameroon, *GSTe2* haplotype dataset for Africa-wide analysis and *Rdl* haplotype dataset, respectively. These models were then used to build the respective ML tree using MEGA 10.1.6 with 500 bootstrap replications for the robustness of the trees. Neighbour-Joining trees were also constructed with pairwise *G_ST_* genetic distances [59] between subpopulations still in MEGA 10.1.6. 

## 3. Results

### 3.1. Mosquito Species Composition

A total of 4911 female mosquitoes were collected across the study localities during the four seasons of collections (Figure S1 and Table S1). Four mosquito genera were found present in the Mount Cameroon region, these are: 13.5% *Mansonia* spp., 19.9% *Culex* spp., 1.6% *Aedes* spp. and 65% *Anopheles* spp. The highest number of mosquitoes was collected from Mutengene (*n* = 2094) and the least number from Likoko (*n* = 319). A significant difference based on Chi-square test was noted in mosquito distribution between all four sites (*p* < 0.0001).

Morphological identification of the 3194 females *Anopheles* revealed that they mostly belong to two major malaria vector species, *An. gambiae* complex (86%) and *An. funestus* group (12.2%) and three secondary vector species (1.6% *An. hancocki*, 0.2% *An. nili* s.l. and 0.03% *An. ziemanni*) (Figure S2), with a significant difference noted in the frequency distributions of *Anopheles* sp. between localities (*p* < 0.0001). PCR-species identification performed from 3135 females morphologically identified as *An. gambiae* s.l. and *An. funestus* s.l. revealed that three *An. gambiae* siblings coexist in the study area: *An. coluzzii* (44.7%), *An. gambiae* (28.6%) and *An. melas* (12%), in addition to 23 hybrids *An. coluzzii* × *An. gambiae* (0.7%). Conversely, all *An. funestus* s.l. individuals were identified as *An. funestus* s.s.

### 3.2. Parity and Plasmodium Infection Rates

Of the 3135 female *Anopheles* identified as either *An. funestus* s.s. or belonging to the *An. gambiae* complex, the tracheal filaments of 2490 (79.4%) were analysed for physiological age determination (parity rate). Of these, 2004 (80.5%) dissected females were found parous (Figure S3). The highest parity rate was observed in *An. melas* (87%; 301/346), whereas 82.5% (558/676) *An. gambiae*, 81% (243/300) *An. funestus* s.s., 77.2% (885/1147) *An. coluzzii* and 76.2% (16/21) *An. coluzzii* × *An. gambiae* hybrids were detected parous. Parity rates showed significant differences between dissected mosquito species (*p* = 0.003) and between the surveyed localities except for the lowland Tiko and Likoko highland (*p* = 0.764).

Similarly, a total of 2746 *An. gambiae* s.l. specimens and 364 *An. funestus* s.s. were screened for *P. falciparum* (falcip+) and *P. ovale*/*P. vivax*/*P. malariae* (OVM+) using TaqMan assay on genomic DNA extracted from whole mosquitoes (Figure S3). An overall infection rate (IR) of 2.35% (73/3110) was obtained, with *An. funestus* s.s. being more infected (4.67%, 17/364), while 0.79% *An. melas* (3/381), 1.61% *An. coluzzii* (23/1428), 3.17% *An. gambiae* (29/914) and 1/23 *An. coluzzii* × *An. gambiae* hybrids were tested positive for *Plasmodium* spp. infection. Of the 73 *Anopheles* mosquitoes tested positive for *Plasmodium* spp. infection, 57 mosquitoes were falcip+ (78.1%), 14 OVM+ (19.2%) and 2 were Pf/OVM+ (2.7%). The multiplex PCR assays performed with infected mosquitoes confirmed TaqMan results and determined that the 16 OVM+ individuals specifically correspond to 15 *P. malariae* and one *P. vivax*. Mosquitoes presenting mixed infection Pf/OVM+ were mix-infected with *P. falciparum* and *P. malariae*. A significant difference was noted between the infection rates of the different *Anopheles* species tested (*p* = 0.04). However, no significant differences were observed in mosquito’s infection rates between Tiko/Meanja (*p* = 0.203) and Mutengene/Likoko (*p* = 0.863).

### 3.3. Detection of Mutations Associated with DDT and Dieldrin Resistance in An. funestus s.s. Mosquitoes

**Figure 2:**
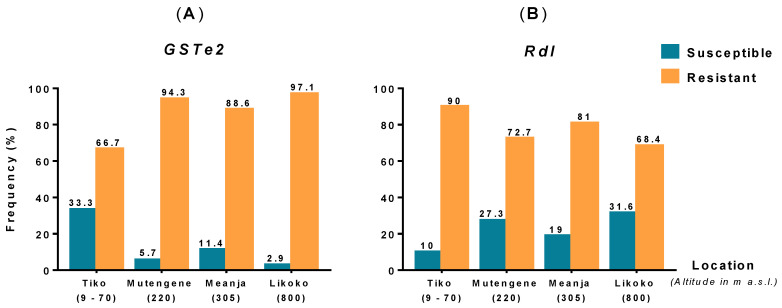
Frequency distribution of *GSTe2* (**A**) and *Rdl* (**B**) mutations in *An. funestus* s.s. populations from the localities surveyed.

The presence of *GSTe2* and *Rdl* mutations was investigated respectively in 339 and 218 *An. funestus* s.s. specimens across the study area. Genotyping results showed high frequencies of *GSTe2*^R^ (66.7–97.1%) (Figure 2A) and *Rdl*^R^ (68.4–90%) (Figure 2B) resistance mutations in *An. funestus* s.s. populations, with an overall resistant genotype estimated at 93.5% (317/339) for the *GSTe2* gene and 74.3% (162/218) for the *Rdl* gene. It was interesting to note that land elevation seems to contrarily influence the frequency of both mutations across the study area. In fact, the frequency of *GSTe2* resistance increased with altitude, whereas that of *Rdl* decreased with altitude. Conversely, while the frequency of *GSTe2* susceptible specimen decreased with altitude, the proportion of dieldrin susceptible mosquitoes increased with climb in altitude. However, no significant differences based on Chi-square test were noted in the overall distribution of both markers in *An. funestus* s.s. populations across the study area (*GSTe2 p* = 0.446 and *Rdl p* = 0.543).

**Figure 3:**
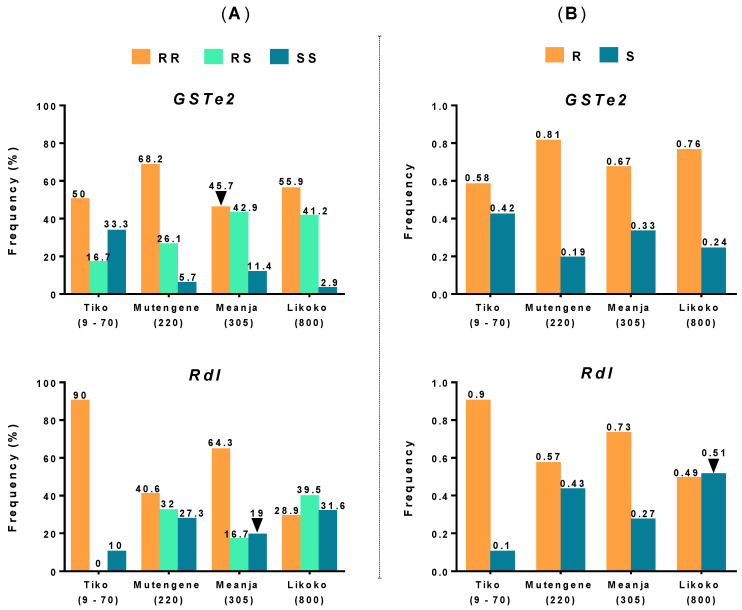
Genotype (**A**) and allele (**B**) frequency distributions of *GSTe2* and *Rdl* mutations in *An. funestus* s.s. populations for the different localities surveyed (altitude in m a.s.l.).

Specifically, *GSTe2* mutation had an overall frequency of 64.3% (218/339) for homozygote-resistant mosquitoes (RR), 29.2% (99/339) heterozygotes (RS) and 6.5% (22/339) homozygotes susceptible (SS). A predominance of homozygote-resistant genotypes was observed throughout the study area being 50% (3/6) in Tiko, 68.2% (180/264) in Mutengene, 45.7% (16/35) in Meanja and 55.9% (19/34) in Likoko (Figure 3A). Increased frequencies of *GSTe2* heterozygotes were noted with the climb in altitude, whereas the occurrence of susceptible mosquitoes decreases with land elevation. Meanwhile, for *Rdl* mutation, although homozygotes-resistant mosquitoes almost dominated across the study area (Tiko: 90% (9/10), Mutengene: 40.6% (25/128), Meanja: 64.3% (27/42), Likoko: 28.9% (11/38)), the frequencies of heterozygotes and susceptible mosquitoes followed reverse tendencies to those of *GSTe2*. For both makers, Chi-square test statistical analysis of genotype frequency distributions between localities showed significant differences except in Tiko/Meanja (*GSTe2*: *p* = 0.273, *Rdl*: *p* = 0.244), Mutengene/Likoko (*GSTe2*: *p* = 0.17, *Rdl*: *p* = 0.423) and Meanja/Likoko (only *GSTe2*: *p* = 0.354).

Analysis of the allele frequencies of *GSTe2* and *Rdl* mutations in *An. funestus* s.s. populations from the four localities revealed the same pattern as those obtained with genotyping results (Figure 3B). *GSTe2*^R^- and *Rdl*^R^-resistant alleles were predominantly represented in almost all the collection sites, except in Likoko, where the *Rdl*^S^ susceptible allele was predominant (with a frequency of 0.51). However, both markers showed contrary evolution in their alleles’ frequency distributions when considering land elevation. For resistant alleles, while the frequency of *GSTe2*^R^ seemed to increase with altitude, *Rdl*^R^ frequency decreased with altitude. Meanwhile, the frequencies of susceptible alleles portrayed a reverse tendency to those observed with resistant ones.

### 3.4. Analysis of the Polymorphism of GSTe2 and Rdl Genes across Mount Cameroon An. funestus s.s. Populations

#### 3.4.1. Sequence Analysis of Full Length *GSTe2* and *Rdl* Genes

*GSTe2* and *Rdl* full length fragments were successfully amplified in 36 and 34 *An. funestus* s.s. genomic DNA samples, respectively, with specific primers for both mutations (Table S2). The alignment and comparison of sequences obtained with that referenced in Genbank confirmed the presence of glutathione S-transferase Epsilon 2 protein of *An. funestus* (AHC31021.1) and exon 7 encoding the M2 transmembrane domain region of *An. funestus* GABA-receptor gene (AZB49494.1), respectively, for *GSTe2* and *Rdl* amplicons.

**Figure 4:**
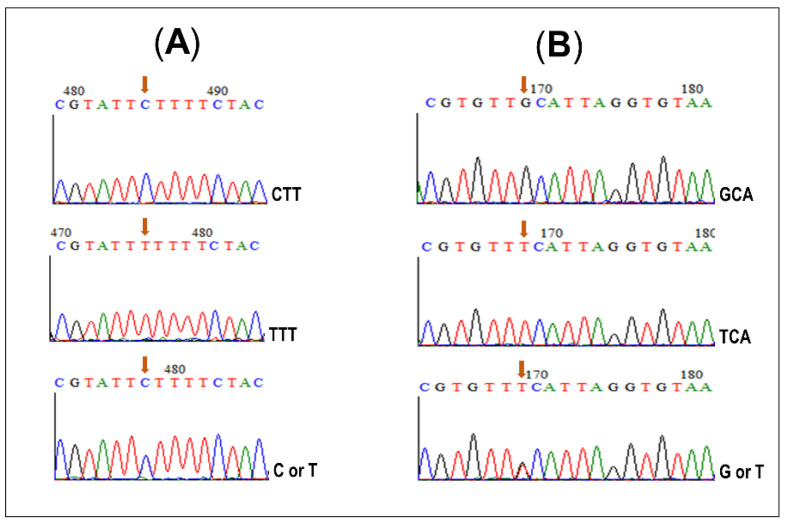
Sequence chromatograms of a 729 bp fragment in *GSTe2* gene (**A**) and a 1006 bp fragment of the GABA-receptor gene (**B**); for susceptible (top), homozygote-resistant (middle) and heterozygote (bottom) *An. funestus* s.s. The polymorphic site is indicated by the red arrow.

A point mutation (CTT to TTT) at position codon 119, inducing an amino acid change of leucine to phenylalanine (L119F) and which confers resistance to pyrethroids in *An*. *funestus* s.s. mosquitoes, was observed in all *GSTe2*-resistant samples of Mount Cameroon (Figure 4A). Likewise, the GCA (alanine) to TCA (serine) mutation at position codon 296 (A296S) was observed in *An. funestus* s.s. dieldrin-resistant samples (Figure 4B).

#### 3.4.2. Haplotype Distribution of the *GSTe2* Gene

**Figure 5:**
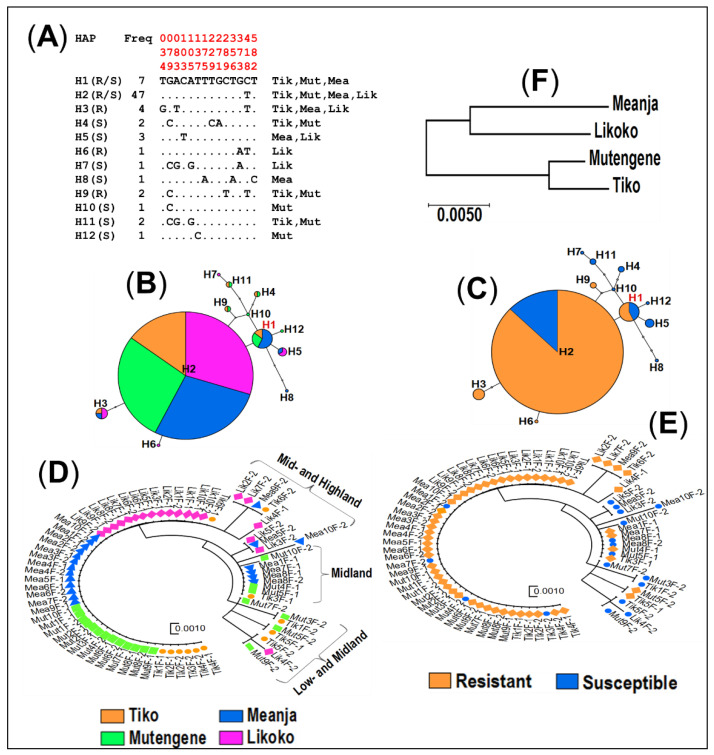
Genetic diversity and polymorphism patterns of *GSTe2* DNA sequences across Mount Cameroon. (**A**) Haplotype diversity patterns of the 729bp GSTe2 fragment in Tiko (Tik), Mutengene (Mut), Meanja (Mea) and Likoko (Lik); H = haplotype; R = resistant; S = susceptible; polymorphic sites are in red. TCS haplotype networks showing haplotype’s generation within GSTe2 gene in *An. funestus* s.s with respect to localities (**B**) and allelic profiles (**C**): haplotypes are presented in circular shape scaled to reflect their respective frequencies; Ancestral haplotype in red. Maximum likelihood phylogenetic trees of *GSTe2* DNA sequences among localities (**D**) and allelic profiles (**E**) showing an apparent separation between susceptible samples and resistant ones. (**F**) Neighbour-joining tree of the genetic distances showing a genetic relatedness to the landscape along the Mountain altitudinal transect with Tiko and Mutengene (9–220 m a.s.l., both located on the southwest edge) clustering together more than Likoko (800 m a.s.l., west edge) and Meanja (305 m a.s.l., eastern edge).

A total of 14 variable or polymorphic sites, which led to the formation of 12 haplotypes, were observed within the 729 bp fragment of *GSTe2* gene of *An. funestus* s.s. moquitoes across the Mount Cameroon region (Figure 5A and Table 1). The nucleotide sequences of the haplotypes were submitted to Genbank (accession numbers: MN562756, MN562757, MN562760, MN562764–MN562766, MN562768–MN562771, MN562774 and MN562775).

Overall, the *GSTe2* polymorphism level was average (haplotype diversity: Hd = 0.56). The number of haplotypes (h) and its associated diversity index (Hd) seemingly decreased (0.68–0.51) from the lowland Tiko (9–70 m a.s.l.) to Likoko situated at the highest altitude (800 m a.s.l.). Samples carrying the L119 susceptible allele were found highly polymorphic (h = 9 and Hd = 0.91) as compared to 119F-resistant allele carriers (h = 5 and Hd = 0.36).

Haplotype networks (Figure 5B,C) were built using TCS software. Haplotype network representation with respect to collection sites (Figure 5B) showed the presence of a unique major haplotype (H2: 47/72 sequences) distributed from lowland to highland across the study area. The ancestral haplotype (H1) appeared in lowland (Tiko: 9–70 m a.s.l.; one sequence) and midlands (Mutengene: 220 m a.s.l., two sequences and Meanja: 305 m a.s.l., four sequences) but not in highland (Likoko: 800 m a.s.l.). Three haplotypes (H4, H9 and H11) were half-shared between Tiko and Mutengene located at 9 to 220 m a.s.l.; another haplotype (H3) was concomitantly found in both Tiko (9–70 m a.s.l.; one sequence), Meanja (305 m a.s.l.; one sequence) and Likoko (800 m a.s.l.; two sequences), and one other (H5), only found in Meanja (one sequence) and Likoko (two sequences). Out of the 12 haplotypes identified, five occurred as singletons and were distributed as follows: two (H10 and H12) in Mutengene (220 m a.s.l.), one (H8) in Meanja (305 m a.s.l.) and two (H6 and H7) in Likoko (800 m a.s.l.) (Figure 5A).

Comparatively, haplotype network representation with respect to the allele type (either susceptible or resistant) carried by the sequences analysed (Figure 5C) showed that out of the 12 haplotypes recorded, only two (H1 and H2) were common to both L119 and 119F carriers, whereas three haplotypes (H3, H6 and H9) were strictly found in 119F carriers and the remaining seven haplotypes (H4, H5, H7, H8, and H10–H12) appeared only in L119 carriers. It was noted that common haplotypes to both L119 and 119F carriers were predominantly found in resistance sequences (H1: 4 sequences of 119F and 3 sequences of L119; H2: 41 sequences of 119F and 6 sequences of L119). Additionally, out of the five haplotypes which appeared as singletons, four were strictly associated to L119 carriers (H7, H8, H10 and H12), whereas only one haplotype (H6) originated from a 119F carrier.

Evidence of selection acting on *GSTe2* gene could be noted as 119F-resistant allele carriers that exhibited low diversity parameters (h = 5, Hd = 0.36 and π = 0.001), unless negative and non-significant values were obtained from Tajima D and Fu and Li F * neutrality tests (Table 1).

*Gste2* gene Africa-wide analysis.

The alignment of the 72 GSTe2 sequences analysed in the present work with 90 other sequences [17] carrying the same mutation and which were previously reported in Africa (Malawi: 18 sequences, Mozambique: 10 sequences, Uganda: 10 sequences, Benin: 24 sequences, Ghana: 16 sequences and North Cameroon: 12 sequences) revealed the presence of a total of 58 polymorphic sites, resulting in the formation of 53 haplotypes across Africa (Figure S4 and Table S3). The most prevalent haplotype H18 (48/162 sequences) appeared in both L119-susceptible (8 sequences) and 119F-resistant (40 sequences) allele carriers. It was shared by Mount Cameroon (8 sequences of L119 and 39 sequences of 119F) and Ugandan (one 119F sequence) populations (Figure S5). Another major haplotype H3 (16/162 sequences) was found in both L119 (11 sequences) and 119F (five sequences) allele carriers of Mount Cameroon (two L119 and five 119F sequences), Malawi (six L119-sequences), Mozambique (two L119-sequences) and Uganda (one L119-sequence); whereas two minor haplotypes still observed in L119 and 119F allele carriers were shared by Mount Cameroon/Malawian samples (H4: one L119 sequence from Malawi and one 119F sequence from Mount Cameroon-Mutengene) and another was only noted in the Mount Cameroon area (H50: one L119-Mutengene sequence and one 119F-Tiko sequence). TCS haplotype networks also showed the presence of a major resistant haplotype (H26: 32/162 sequences) restricted to West and Central African populations (22 in Benin, 5 in Ghana, 4 from Mont Cameroon and 1 from North Cameroon). Overall, a high proportion of singletons was noted (39/53 haplotypes), being mostly L119-suceptible allele carriers (30/39 haplotypes), thus suggesting a high polymorphism with reduced numbers of mutational steps, in addition to a signature of selection within the *GSTe2* gene across some *An. funestus* African populations where a GSTe2-based resistance to pyrethroids/DDT has been reported (haplotype diversity, Hd = 0.86; nucleotide diversity π = 0.005; Tajima D = −2.03 with *p* < 0.05) (Figures S4 and S5 and Table S3). 

The construction of maximum-likelihood (ML) phylogenetic trees revealed that haplotypes cluster according to allelic profiles and irrespectively of their geographical origin. Rather, a neighbour-joining (NJ) distance tree clustered Mount Cameroon alongside north Cameroon *An. funestus* s.s. population, whereas the east–south (Uganda–Malawi and Mozambique) and west (Benin and Ghana) African populations formed separated clusters (Figure S5).

#### 3.4.3. Haplotype Distribution of the GABA-Receptor Gene across Mount Cameroon Populations of *An. funestus* s.s.

**Figure 6:**
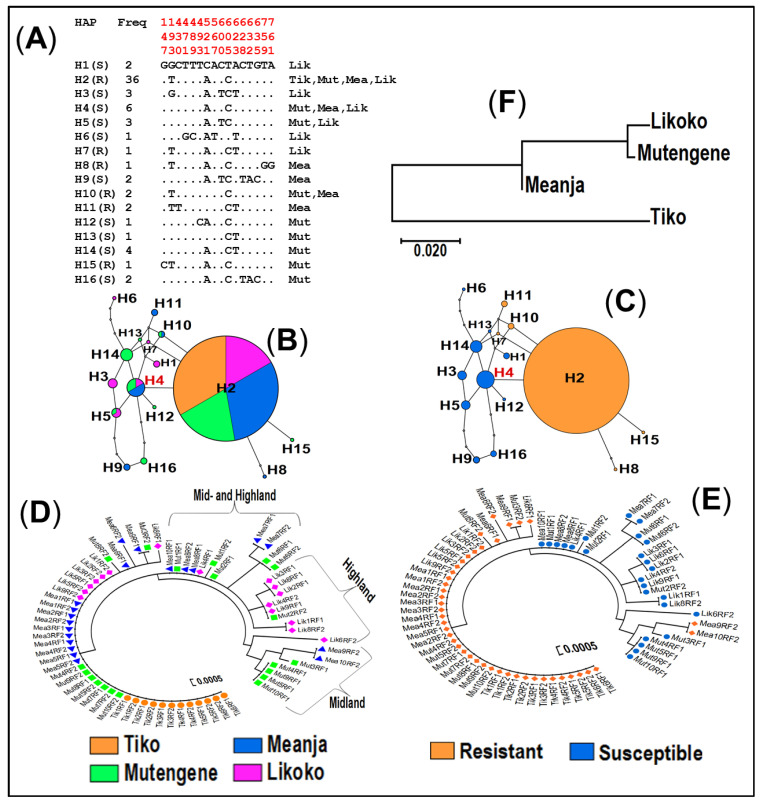
Genetic diversity and polymorphism patterns of the GABA-receptor gene across the Mount Cameroon area. (**A**) Haplotype diversity patterns of a 1006 bp fragment of the GABA-receptor gene in Tiko (Tik), Mutengene (Mut), Meanja (Mea) and Likoko (Lik); H = haplotype; R = resistant; S = susceptible; polymorphic sites are in red. TCS haplotype networks showing a high polymorphism of the GSTe2 gene in *An. funestus* s.s with a reduced number of mutational steps between haplotypes with respect to localities (**B**) and allelic profiles (**C**); haplotypes are presented in circular shape scaled to reflect their respective frequencies; ancestral haplotype is in red. Maximum likelihood phylogenetic trees of *Rdl*-DNA sequences among localities (**D**) and allelic profiles (**E**) showing a marked separation between A296 and 296S allele carriers. (**F**) Neighbour-joining tree of the genetic distances showing an apparent genetic relatedness associated with the altitude along Mount Cameroon with Tiko being genetically differentiated from other localities.

Analysis of 68 sequences for the 1006 bp fragment of the GABA-receptor gene (accession numbers: Genbank MN562780–MN562795) showed the presence of 16 polymorphic sites and the formation of an equal number of haplotypes (Figure 6A and Table 2). Genetic variability parameters with respect to allelic profiles and among the four tested *An. funestus* s.s. populations showed that the high number of haplotypes occurred in A296-susceptible allele carriers (h = 10, with an equivalent high haplotype diversity Hd = 0.90) and for the localities of Mutengene (at 220 m a.s.l., h = 9 and Hd = 0.85) and Likoko (at 800 m a.s.l., h = 7 and Hd = 0.83), while the lowest number of haplotypes was found in Tiko (h = 1, Hd = 0.00) at the lowest base of Mount Cameroon (9–70 m a.s.l.) with no polymorphic site detected.

The most common haplotype (H2: 36/68 sequences) was found to be carrying the 296S-resistant allele and was distributed throughout the study area (Figure 6A–C), unlike the ancestral haplotype (H4: 6/36 sequences), which carried the A296-susceptible allele and was distributed at low (Tiko: 9–70 m a.s.l.) and mid (Mutengene: 220 m a.s.l.; Meanja: 305 m a.s.l.) altitudes but not in highland (Likoko: 800 m a.s.l.). Approximately one third of the haplotypes (6/16) appeared as singleton in A296 (H6, H12 and H13) and 296S (H7, H8 and H15) allele carries, and were identified from midland (Mutengene: H12, H13 and H15; Meanja: H8) to highland (Likoko: H6 and H7).

Overall, there were negative and non-significant values of the selection test from Tajima D and Fu and Li F * (Table 2). However, the positive but not significant F * in Meanja (F *: 0.04), in addition to the low values of haplotype and nucleotide diversity recorded in 296S-resitant allele carriers (Hd = 0.30 and π = 0.0005, respectively) could be indicators of an ongoing selection acting on the GABA receptor gene within the Mount Cameroon *An. funestus* s.s. populations.

### 3.5. Population Structure at GSTe2 and Rdl Mutations in An. funestus s.s. across Mount Cameroon

Analysis of the genetic structure at the 729 bp *GSTe2* fragment supported the genetic variability observed between low- (Tiko), mid- (Mutengene and Meanja) and highland (Likoko). Construction of the maximum likelihood (ML) phylogenetic tree with respect to localities (Figure 5D) showed the presence of a major consensus cluster across Mount Cameroon, in addition to three apparent separated clusters formed by haplotypes of low- to midland, midland and mid- to highland. Conversely, the construction of the ML tree with respect to allelic profile (Figure 5E) highlighted the reduced diversity of 119F-resistant allele carriers across the study area. The patterns of clustering according to locations was further supported by low values of genetic differentiation estimates (−0.0006 ≤ K_ST_ ≤ 0.010; all not significant) obtained between the four localities tested and the Nm gene flow index, which showed a marked genetic closeness between Tiko/Mutengene (low- and midland) and Meanja/Likoko (mid- and highland), as illustrated on the NJ tree of genetic distances (Figure 5F and Table 3).

Similarly, analysis of the ML phylogenetic tree at the GABA-receptor for *Rdl* mutation based on localities highlighted the presence of a main consensus cluster across Mount Cameroon and three other apparent clusters generated by midland, mid-/highland and highland haplotypes (Figure 6D). Conversely, the ML tree with respect to allelic profiles highlighted the marked the reduced diversity of 296S-resistant allele carriers (Figure 6E). The construction of a NJ tree of genetic distance (Figure 6F) revealed that the lowland Tiko clusters separately from other localities aligned following the same altitudinal transect (Mutengene and Likoko) or situated eastward of the mountain (Meanja), all at a higher elevation level (200–800 m a.s.l.). This latter observation was further supported by the consistency of high and significant values of genetic differentiation estimates observed between Tiko and the other three localities (0.037 < K_ST_ < 0.158) (Table 4).

## 4. Discussion

The implementation of effective insecticide resistance management strategies relies on the good understanding of the direction and speed of spread of resistance alleles among mosquito populations. This study assessed the influence of Mount Cameroon on the spread of both GST-mediated metabolic resistance and Rdl-based target-site resistance among population of *An. funestus* s.s. malaria vector.

Results from this study correlates with observations from previous reports [39,60] indicating the predominance of *Anopheles* mosquitoes within the overall mosquito fauna found in the Mount Cameroon region. *Anopheles* mosquitoes were collected throughout the study area (from lowland to highland), and a total of seven *Anopheles* species were identified; these included sibling species (*An. coluzzii*, *An. gambiae* and *An. melas*) of the *An. gambiae* sensu lato (s.l.) complex, *An. funestus* s.s., *An hancocki*, *An. nili* and *An. ziemanni*. The same species have already been found in other elevated areas, such as those of western [14] and north-western Cameroon [61]. *Anopheles funestus* s.s. appeared as the second dominant vector species after *An. gambiae* s.l., confirming previous reports in these sites [13,62]. Overall, *An. funestus* s.s. abundance increased from the coastal lowland Tiko to Likoko highland, with relatively high abundances observed in mid-altitude areas (Mutengene and Meanja), probably due to its larval habitat preference. Breeding sites of *An. funestus* s.s. are limited to large permanent waters with aquatic vegetation [63,64,65], habitats which are quite abundant in Mutengene and Meanja areas [62].

In the Mount Cameroon area, the *An. funestus* s.s. vector seemed to play a major role in malaria transmission as compared to *An. gambiae* s.l. vectors, especially during the dry season months (November–February). This attested to the role of *An. funestus* s.s. in bridging the gap of malaria transmission between rainy and dry seasons [63], and compensating the lack of malaria transmission induced by microclimatic conditions in highlands [14,39]. Moreover, high parity rates obtained could be signs of an increased life expectancy of *Anopheles* vectors across the region. Similar observations were made in previous studies, thus implying a greater chance for possible vector-host contact and ultimately the transmission of *Plasmodium* parasite for competent vectors [39,61].

The genotyping of GSTe2-based DDT resistance maker showed the presence at high frequencies of *GSTe2^R^* genotypes and alleles in all *An. funestus* s.s. populations tested throughout the Mount Cameroon altitudinal gradient (7–800 m a.s.l.). This presence of GST-based metabolic resistance was further confirmed after sequencing by the detection of a single substitution of nucleotide at position 119, inducing an amino acid change of leucine (CTT) to phenylalanine (TTT) [20]. GST-based metabolic resistance is common in a number of anopheline species, including *An. gambiae* s.l. [66,67] and *An. funestus* s.s. [16,49,68], reflecting the heavy use of DDT and pyrethroids for malaria control over several decades [7,69]. In Cameroon, the intensive use of these insecticide classes to control agriculture pests, especially in agroecosystems such as those found in the Mount Cameroon region (especially Tiko, Mutengene and Meanja in this study), may have contaminated mosquito breeding sites, thus exerting significant and constant selection pressure on *Anopheles* populations [60].

Similarly, *An. funestus* s.s. populations from Mount Cameroon exhibited high levels of mutations in the *Rdl* gene, which encoded for the GABA-receptor subunit. Dieldrin resistance have already been documented in insect species [70] including the malaria vectors *An. stephensi* [71], *An. gambiae* s.l. [72] and *An. funestus* [15]. In Cameroon, high frequencies of *Rdl* mutation have also been reported in areas other than the Mount Cameroon region [16,50,73,74], despite the fact that cyclodienes are no longer used for control programs. Moreover, observations made in this study raise concerns about the use of agrochemicals targeting the GABA-receptor in the agricultural environment. Thus, understanding the factors which could possibly explain the persistence of this resistance in *An. funestus* populations are greatly needed in order to get insights on resistance management.

Thirty-nine percent of *An. funestus* s.s.-tested specimens were found carrying both *GSTe2^R^* and *Rdl^R^* alleles simultaneously, confirming an earlier report [16], which had identified multiple resistance markers in *An. funestus* s.s. populations from Gounougou, in the Northern part of the country. The extensive use of agrochemicals for crop protection coupled to long lasting insecticidal nets (LLINs), massively distributed since 2011 for public health activities through the National Malaria Control Program of the Ministry of Public Health, might have also greatly influenced the selection of these mutations in vector populations. However, investigations are required on the frequency and distribution of metabolic and target-site resistant alleles with respect to altitude, as well as on the validation of pyrethroids resistance in *An. funestus* s.s. populations from thorough fully representative sites across this domain, which is highly favourable for agricultural activities. These will represent an added value in further understanding the linkage between resistance markers and the use of agrochemical and malaria public health activities.

Our results showed substantial variation in GSTe2-based and dieldrin resistance trends within the Mount Cameroon domain. Interestingly, *GSTe2^R^* allelic frequencies increased with land elevation, whereas *Rdl^R^* frequencies decreased with an increase in altitude. These observations suggest that altitude could positively favour the establishment of *An. funestus* GSTe2-resistant populations from mid- (Mutengene and Meanja) to highland (Likoko) areas, unlike dieldrin-resistant populations, which seemed to be more adapted to Tiko, the lowest elevated site of the Mount Cameroon region as investigated in this study. Land elevation had previously been reported as an important influential predictor of the increase in pyrethroid resistance in the *An. gambiae* species complex in west Africa, though not in east Africa [75]. This study highlights some uncertainties of the potential influence of altitude on the maintenance of insecticide resistances in malaria vector populations under specific environmental conditions similar to that of the study area. In such areas, field sampling to measure resistance is the only means of informing resistance management decisions alongside an assessment of the historical and contemporary role of pesticide usage and the role of public health insecticide use in the development of insecticide resistance in malaria vectors, as previously reported [76].

It has been reported that landscape variations are associated with the risk of presence and insecticide resistance for malaria vectors [77,78,79]. Topography and land use influence vector densities, level of exposure to insecticide and resistance development in mosquitoes, especially in areas with combined exposure to insecticide from agricultural and/or vector control activities [80]. Distribution of chromosomal inversions such as those found in resistance to insecticides had already been extensively associated with latitudinal but less frequently with altitudinal changes [81,82,83]; thus, investigations are needed in order to further elucidate the association between altitudinal changes and the geographical distribution of insecticide resistance.

Patterns of genetic differentiation based on *GSTe2* mutation revealed that Tiko (9–70 m a.s.l.) and Mutengene (220 m a.s.l.) populations of *An. funestus* s.s. are genetically differentiated to that of Meanja (305 m a.s.l.) and Likoko (800 m a.s.l.) as they formed a unique cluster compared to others on the neighbour-joining tree of distance. Out of the 12 haplotypes identified, *An. funestus* s.s. populations from Tiko and Mutengene appeared to share six haplotypes, of which three are exclusively found in these localities. The causes of this clustering could be associated with the similar geographical position of both populations around Mount Cameroon (southwestern edge) or the presence of the mountain itself, which affects the population genetic structure and the speed of spread of the *GSTe2^R^* allele between *An. funestus* s.s. populations. Population structure analysis based on *GSTe2* gene support the contrast in resistance patterns between *An. funestus* s.s. populations and further suggest the presence of barriers to gene flow between these populations. Similar geographical barriers to the spread of resistance alleles has been mentioned for other resistance makers, such as P450-based metabolic resistance in *An. funestus* [84] or knock-down resistance (*kdr*) mutations in *An. gambiae* [9].

Comparatively, genetic variability patterns within the GABA-receptor based *Rdl* mutation showed that *An. funestus* s.s. population from Tiko at the base of Mount Cameroon is more genetically differentiated to mid- and highland vector populations, as it separately clusters to other localities. This strong differentiation observed on *Rdl* mutation in Tiko was confirmed by the high and significant values of K_ST_ statistics of the genetic differentiation obtained for the *An. funestus* s.s. population from Tiko. Hence, it can be hypothesised that the presence of Mount Cameroon (mountain) influences the contrast in *Rdl* resistance patterns between populations of *An. funestus* s.s. in the study area, thus suggesting the presence of barriers of gene flow between *An. funestus* s.s. populations. This is further supported by the reduced genetic diversities parameters (h and Hd), the positive value of the Fu and Li F* index and the significant K_ST_ statistics obtained in Meanja midland (eastern edge of Mount Cameroon) compared to the mid- (Mutengene) and highland (Likoko) localities of the Great West. Nevertheless, investigations of more vector populations from both sides of Mount Cameroon are needed to validate such hypotheses.

A strong selection process was observed on both 119F and 296S resistance allele carriers. This can be seen by the reduced genetic diversity parameters (h, Hd, π and k) with a limited number of mutational steps (polymorphic sites) between haplotypes in resistance allele carriers compared to susceptible allele carriers, which maintained high diversity parameters. The analysis of the ML tree based on allelic profiles further illustrated the reduced diversity of resistance allele carriers in both cases. Furthermore, the most predominant haplotype is found in 85% (GSTe2-based mutation) to 100% (*Rdl* mutation) resistance allele carriers, which is indicative of an ongoing selection on 119F and 296S alleles, contrasting with L119 and A296 susceptible alleles, which maintained a high number of singletons. Similar selection patterns have been observed in P450 [84] and GSTe2 [20] genes in *An. funestus* populations from other African regions. The selection process could be due to intensive use of insecticides through routine integrated control carried out by the National Malaria Control Program in the Mount Cameroon region, particularly in the localities surveyed. In addition, positive selection could also be associated with adaptation of mosquito larval stages to agricultural pesticides and other adverse conditions, such as temperature and landscape [85,86].

Based on the *GSTe2* gene, comparing populations of *An. funestus* s.s. from the Mount Cameroon region with other *An. funestus* African populations revealed that Cameroonian (including Mount Cameroon and North Cameroon) populations of *An. funestus* seemingly present similar patterns of genetic diversity. Despite the presence of the *GSTe2^R^* allele recorded in Mount Cameroon (at high frequencies), high values of genetic diversity parameters were still obtained compared to that of Benin, where a marked reduced diversity was noted. However, the high diversity observed in Mount Cameroon, besides high frequencies of the *GSTe2^R^* allele, reflects a situation of moderate selection of the 119F allele, which could progressively change in the future to become as Benin, where the *GSTe2^R^* allele is nearly driven to fixation due to a greater selection of resistance [20]. The high diversity obtained in Cameroon is further reinforced by the high level of diversity in L119 susceptible allele carriers. Thereby, if selective pressure continues to act within *An. funestus* populations, resistance profiles are likely to changes as previously reported for P450-based metabolic resistance, where the CYP6P9a-R allele frequency increased from 7.5% in 2002 to 100% in 2017 due to a scale-up of insecticidal bed-nets [87].

## 5. Conclusions

Broadly, this study highlights for the first time the presence of GSTe2-based metabolic resistance and the GABA-receptor target-site mutation associated to dieldrin resistance in the malaria vectors *An. funestus* s.s. across the Mount Cameroon domain. Both *GSTe2^R^* and *Rdl^R^* alleles were found with high frequencies in almost all the localities surveyed; however, the speed of spread of these two molecular mechanisms appears to be influenced by the presence of a major mountainous barrier, Mount Cameroon, which contrasts the resistance and diversity patterns of these two genes between populations of *An. funestus* s.s. in the study area. Furthermore, we provide evidence of positive selection occurring on *GSTe2^R^* and *Rdl^R^* throughout the Mount Cameroon region, which, if not adequately monitored, could drive to a fixation in response to a greater selection of resistance in the future. This emphasises the need of molecular studies of multiple collections sites throughout such mountainous landscapes to fully elucidate the role of environmental changes on the acquisition of insecticide resistance in *Anopheles* vector populations and to mitigate against further spread of resistance through the development of new vector management strategies.

## Figures and Tables

**Table 1:** Genetic variability parameters of *GSTe2* full mutation.

Samples	N	S *	h (Hd)	Syn	NSyn	π (k)	D	F *
Per allele								
S	18	11	9 (0.91)	4	2 (D61E, L119F)	0.003 (2.69)	−0.84 ^ns^	−0.70 ^ns^
R	54	6	5 (0.36)	2	1 (L119F)	0.001 (0.60)	−1.36 ^ns^	−0.27 ^ns^
Per locality (altitude in m a.s.l.)								
Tiko (9–70)	12	8	6 (0.68)	4	1 (L119F)	0.003 (1.97)	−1.38 ^ns^	−1.77 ^ns^
Mutengene (220)	20	8	7 (0.58)	4	1 (L119F)	0.002 (1.38)	−1.31 ^ns^	−2.13 ^ns^
Meanja (305)	20	7	5 (0.56)	2	2 (D61E, L119F)	0.001 (1.04)	−1.55 ^ns^	−2.54 ^ns^
Likoko (800)	20	7	5 (0.51)	1	1 (L119F)	0.002 (1.32)	−1.40 ^ns^	−0.76 ^ns^
ALL	72	14	12 (0.56)	6	2	0.002 (1.36)	−1.62 ^ns^	−1.02 ^ns^

N = number of sequences (2n); S *, number of polymorphic sites; h, number of haplotypes (Hd = haplotype diversity); Syn, Synonymous mutations; Nsyn, Non-synonymous mutations; π, nucleotide diversity (k = mean number of nucleotide differences); D and F * Tajima’s and Fu and Li’s statistics; ns, not significant; S = susceptible; R = resistant; m a.s.l. = meters above the sea level.

**Table 2:** Genetic variability parameters of *Rdl* full mutation.

Samples	N	S *	h (Hd)	Syn	NSyn	π (k)	D	F *
Per Allele								
S	25	11	10 (0.90)	0	0	0.003 (2.53)	−0.44 ^ns^	−0.53 ^ns^
R	43	6	6 (0.30)	1	0	0.0005 (0.54)	−1.61 ^ns^	−1.65 ^ns^
Per locality (altitude in m a.s.l.)								
Tiko (9–70)	12	0	1 (0.00)	0	0	0 (0.00)	n.a.	n.a.
Mutengene (220)	20	9	9 (0.85)	1	1 (A296S)	0.002 (1.97)	−0.76 ^ns^	−0.39 ^ns^
Meanja (305)	20	10	6 (0.68)	0	1 (A296S)	0.002 (2.00)	−1.02 ^ns^	0.04 ^ns^
Likoko (800)	16	8	7 (0.83)	0	1 (A296S)	0.002 (2.38)	−0.05 ^ns^	−0.21 ^ns^
ALL	68	16	16 (0.71)	1	1	0.002 (1.85)	−1.32 ^ns^	−1.87 ^ns^

N = number of sequences (2n); S *, number of polymorphic sites; h, number of haplotypes (Hd = haplotype diversity); Syn, Synonymous mutations; Nsyn, Non-synonymous mutations; π, nucleotide diversity (k = mean number of nucleotide differences); D and F * Tajima’s and Fu and Li’s statistics; ns, not significant; S = susceptible; R = resistant; m a.s.l. = meters above the sea level.

**Table 3:** Patterns of genetic differentiation between *An. funestus* s.s. populations based on K_ST_ estimates from *GSTe2* mutation with (Nm).

	Tiko	Mutengene	Meanja
Mutengene	−0.027 ^ns^ (0.00)		
Meanja	0.002 ^ns^ (57.05)	0.003 ^ns^ (43.40)	
Likoko	−0.007 ^ns^ (0.00)	0.010 ^ns^ (12.19)	−0.0006 ^ns^ (0.00)

PERMTEST calculates Hudson’s K_ST_ statistic of genetic differentiation. K_ST_ is equal to 12KS/KT, where KS is a weighted mean of K1 and K2 (mean number of differences between sequences in subpopulations 1 and 2, respectively) and KT represents the mean number of differences between two sequences regardless of their subpopulation; ns, not significant.

**Table 4:** Patterns of genetic differentiation between *An. funestus* s.s. populations based on K_ST_ estimates from *Rdl* mutation with (Nm).

	Tiko	Mutengene	Meanja
Mutengene	0.125 ** (1.24)		
Meanja	0.037 * (2.08)	0.0098 ^ns^ (15.82)	
Likoko	0.158 ** (1.16)	0.007 ^ns^ (25.82)	0.035 * (14.54)

PERMTEST calculates Hudson’s K_ST_ statistic of genetic differentiation. K_ST_ is equal to 12KS/KT, where KS is a weighted mean of K1 and K2 (mean number of differences between sequences in subpopulations 1 and 2, respectively) and KT represents the mean number of differences between two sequences regardless of their subpopulations. *, 0.01 < *p* < 0.05; **, 0.001 < *p* < 0.01; ns, not significant.

## Supplementary Materials

The following are available online at https://www.mdpi.com/2073-4425/11/12/1492/s1, Figure S1. Mosquito genera identified at each surveyed locality, Figure S2. Female’s anophelines species composition across the study area, Figure S3. Parity and *Plasmodium* spp. infection rates within *An. gambiae* species complex and of *An. funestus* s.s., Figure S4. Nucleotide sequences of the 53 GSTe2 haplotypes identified across Africa, Figure S5. Africa-wide diversity pattern of a 729 bp fragment of the GSTe2 gene, Table S1: Detailed period (in years) of collections, Table S2: List of primers used in this study, Table S3: Genetic diversity parameters of *GSTe2* gene across Africa.
