# Supplementary material for: Influence of a Major Mountainous Landscape Barrier (Mount Cameroon) on the Spread of Metabolic (GSTe2) and Target-Site (Rdl) Resistance Alleles in the African Malaria Vector Anopheles funestus
Source: Genes (Basel). 2020 Dec 11;11(12):1492. doi: 10.3390/genes11121492 (PMC7764057; doi:10.3390/genes11121492)
Supplement: Supplementary file 1 [file genes-11-01492-s001.zip › Table S1.pdf]

Table S1: Detailed period (in years) of collections.

Localities*	Seasons			
	Peak dry season	Mild rainy season	Peak rainy season	Mild dry season
Tiko	2014	2010	2013	2013
Mutengene	2011	2011	2011	2011
Meanja	2012	2010	2012	2012
Likoko	2014	2010	2013	2013

Peak dry season: January – February;

Mild rainy season: March – April;

Peak rainy season: June – October;

Mild dry season: November – December.

*Depending the surface area of selected localities, two to six neighbourhoods were visited for mosquito collections as follows: four (04) neighbourhoods in Tiko (including Likomba), six (06) in Mutengene, two (02) in Meanja and three(03) in Likoko.
