# Supplementary material for: Influence of a Major Mountainous Landscape Barrier (Mount Cameroon) on the Spread of Metabolic (GSTe2) and Target-Site (Rdl) Resistance Alleles in the African Malaria Vector Anopheles funestus
Source: Genes (Basel). 2020 Dec 11;11(12):1492. doi: 10.3390/genes11121492 (PMC7764057; doi:10.3390/genes11121492)
Supplement: Supplementary file 1 [file genes-11-01492-s001.zip › Table S2.pdf]

Table S2: List of primers used in this study.

	Forward primer 5′→3′	Reverse primer 5′→3′	Expected size (bp)
**Genotyping of metabolic resistance**			
GSTe2Full[o]	GGAATTCCATATGACCAAGCTAGTTCTGTACACGCT	TCTAGATCAAGCTTTAGCATTTTCCTCCTT	846
L119F-Sus1[i]	CATTTCTTATTCTCATTTACAGGAGCGTTATC		312
L119F-Res1[i]	CGAGGAATGTCCGATTTTCCGTAGAATA		523
**TaqMan assay for the detection of resistance to dieldrin (Rdl)**			
SerRdl	TCATATCGTGGGTATCATTTTGGCTAAAT	TCGTTGACGACATCAGTGTTGT	
WT2	TTACACCTAATGCAACACG	Probe specific for the wild-type allele A296 labelled with VIC	
Ser	CACCTAATGAAACACG	Probe specific for the mutant allele 296S labelled with FAM	
**Primers used for amplifications of GSTe2 and Rdl for polymorphism analysis**			
GSTe2Full	GGAATTCCATATGACCAAGCTAGTTCTGTACACGCT	TCTAGATCAAGCTTTAGCATTTTCCTCCTT	846
Rdl0mut	TGTCTGTAACCAAATACCTTCTTCT	GATGCTGGGTTCGGTAAGTGT	1216

o: outer primers; i: inner primers; In green is restriction site for *NdeI*, in blue is for *Xba*I.
