# Supplementary material for: Influence of a Major Mountainous Landscape Barrier (Mount Cameroon) on the Spread of Metabolic (GSTe2) and Target-Site (Rdl) Resistance Alleles in the African Malaria Vector Anopheles funestus
Source: Genes (Basel). 2020 Dec 11;11(12):1492. doi: 10.3390/genes11121492 (PMC7764057; doi:10.3390/genes11121492)
Supplement: Supplementary file 1 [file genes-11-01492-s001.zip › Table S3.pdf]

Table S3: Genetic diversity parameters of *GSTe2* gene across Africa.

Location	N	S*	h (Hd)	Syn	NSyn	π (k)	D	F*
Benin	24	1	2 (0.16)	0	1 (N35K)	0.0002 (0.16)	-0.68^{ns}	0.31^{ns}
Ghana	16	19	12 (0.92)	3	2 (L119F, E132K)	0.007 (4.82)	-0.81^{ns}	-0.58^{ns}
Mount Cameroon	72	14	13 (0.57)	6	2 (D61E, L119F)	0.002 (1.36)	-1.62^{ns}	-1.02^{ns}
North Cameroon	12	16	8 (0.85)	6	7 (K79R, K79S, G81S, L119F, T146K, E197Q, G202S)	0.006 (4.26)	-0.85^{ns}	-0.30^{ns}
Malawi	18	22	10 (0.88)	3	8 (L36R, H69P, R110Q, R112P, K128E, N129D, E132K, T146K)	0.007 (5.27)	-0.69^{ns}	-0.08^{ns}
Mozambique	10	13	8 (0.96)	2	0	0.005 (3.36)	-1.47^{ns}	-1.69^{ns}
Uganda	10	17	9 (0.98)	0	3 (D40E, L119F, L175P)	0.009 (6.67)	0.51^{ns}	0.27^{ns}
All	162	58	53 (0.86)	15	18	0.005 (3.72)	-2.03*	-2.09^{ns}

N = number of sequences (2n); S*, number of polymorphic sites; h, number of haplotypes (Hd = haplotype diversity); Syn, Synonymous mutations; Nsyn, Non-synonymous mutations; π, nucleotide diversity (k = mean number of nucleotide differences); D and F* Tajima's and Fu and Li's statistics; ns, not significant; *, $0.01 < p < 0.05$.
