# Supplementary figures and images for: Influence of a Major Mountainous Landscape Barrier (Mount Cameroon) on the Spread of Metabolic (GSTe2) and Target-Site (Rdl) Resistance Alleles in the African Malaria Vector Anopheles funestus
Source: Genes (Basel). 2020 Dec 11;11(12):1492. doi: 10.3390/genes11121492 (PMC7764057; doi:10.3390/genes11121492)

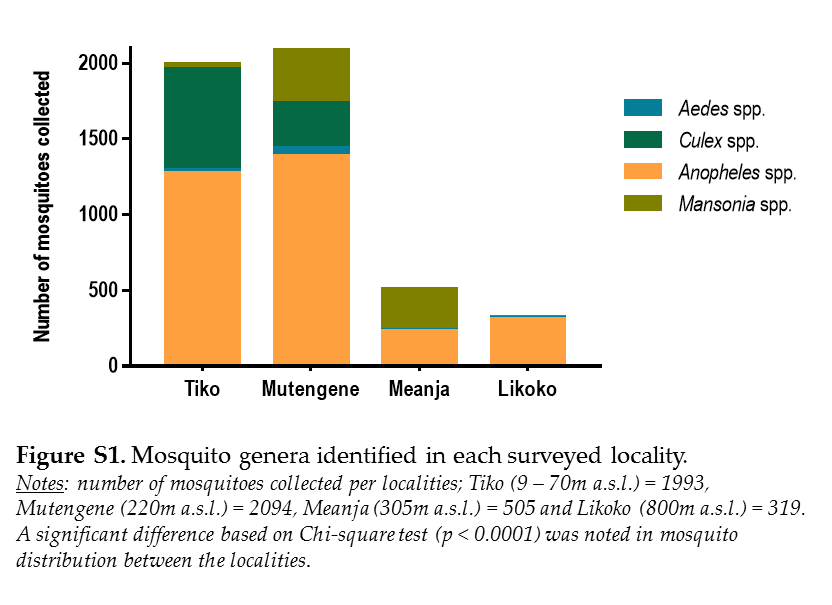

Supplement: Supplementary file 1 [file genes-11-01492-s001.zip › Figure S1.tif]

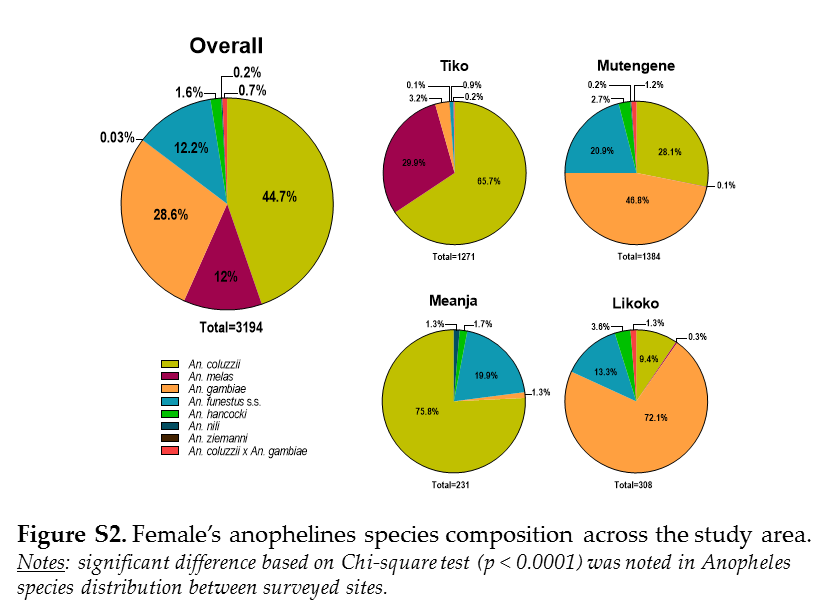

Supplement: Supplementary file 1 [file genes-11-01492-s001.zip › Figure S2.tif]

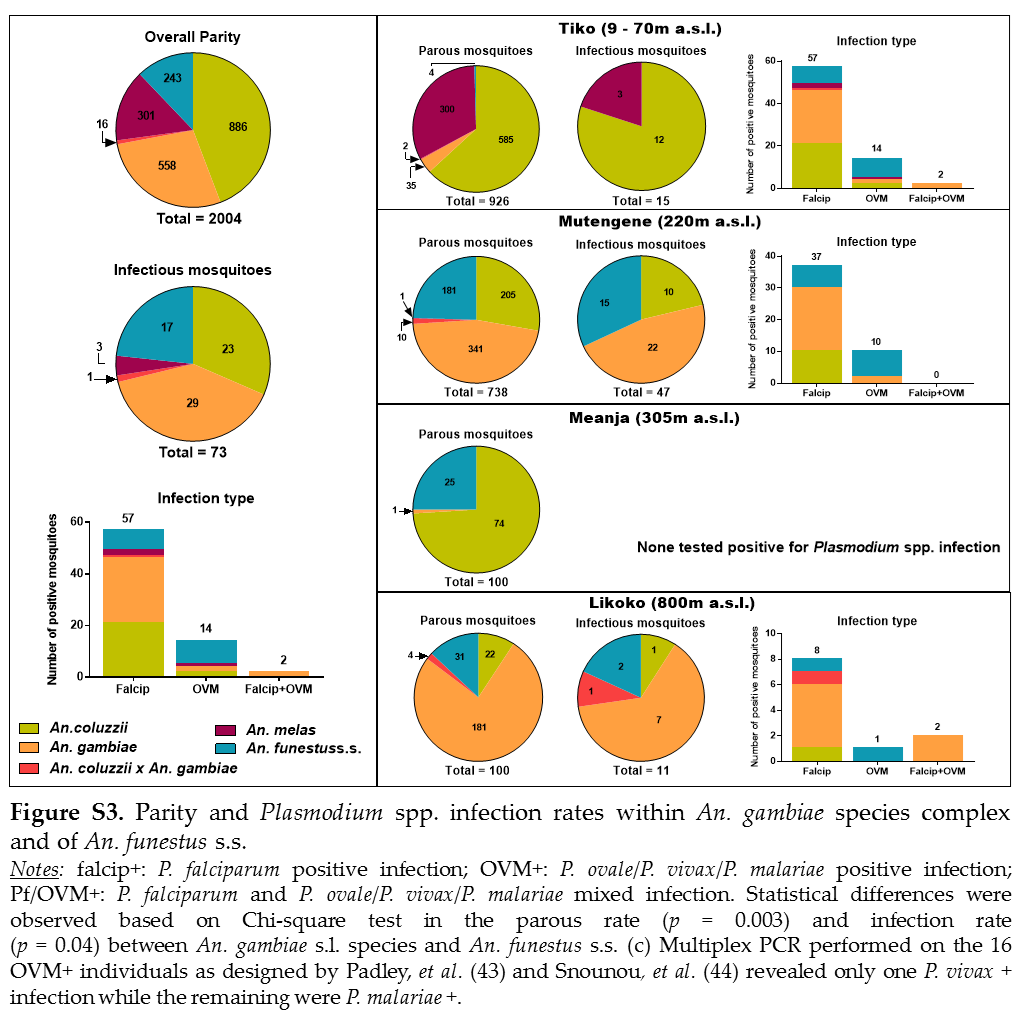

Supplement: Supplementary file 1 [file genes-11-01492-s001.zip › Figure S3.tif]

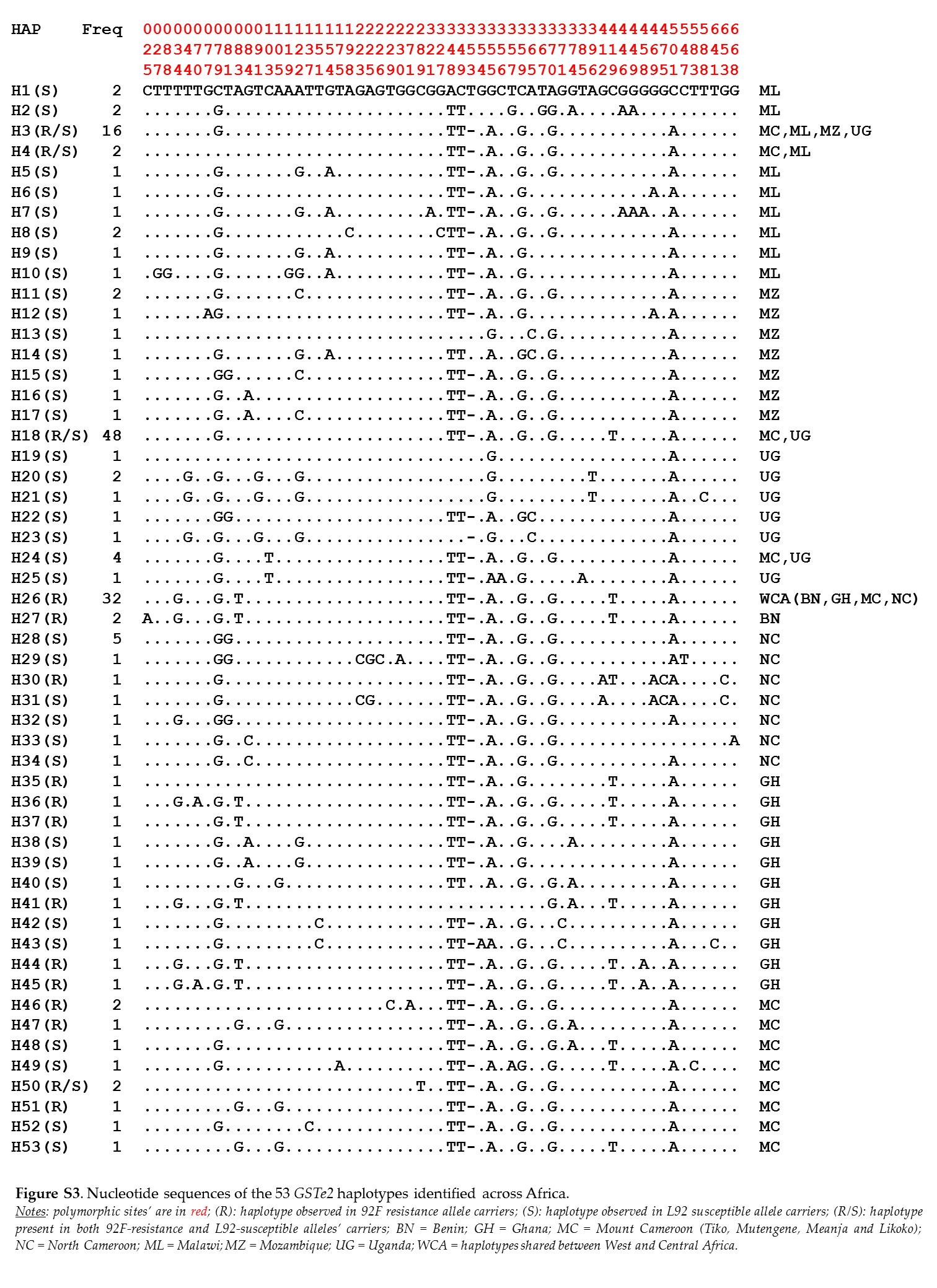

Supplement: Supplementary file 1 [file genes-11-01492-s001.zip › Figure S4.tif]

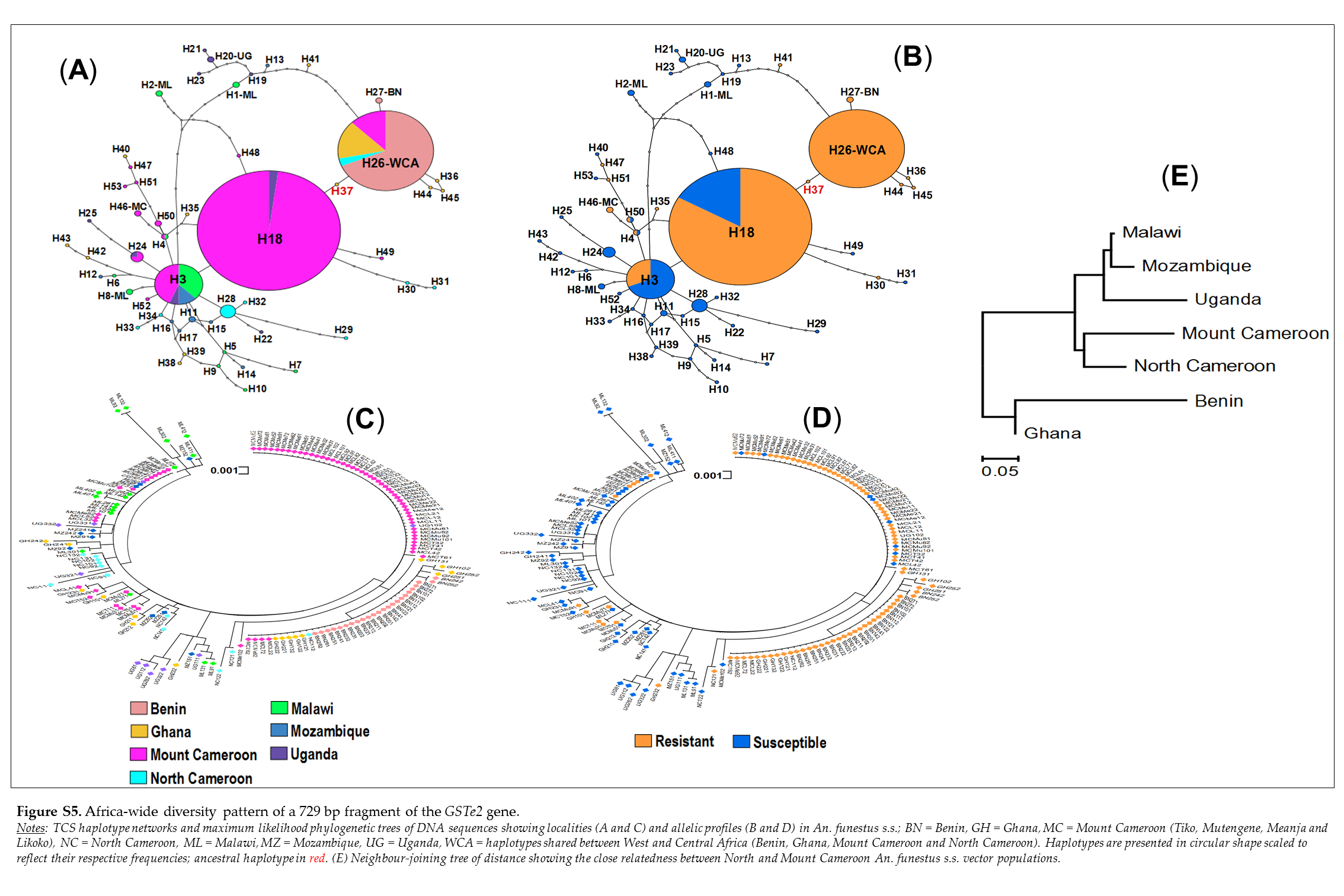

Supplement: Supplementary file 1 [file genes-11-01492-s001.zip › Figure S5.tif]
